# Alarmins, COVID-19 and comorbidities

**DOI:** 10.1080/07853890.2021.1921252

**Published:** 2021-05-27

**Authors:** Eleonora Di Salvo, Mario Di Gioacchino, Alessandro Tonacci, Marco Casciaro, Sebastiano Gangemi

**Affiliations:** a Department of Veterinary Sciences, University of Messina, Messina, Italy; b Center for Advanced Studies and Technology, G. d’Annunzio University, Chieti, Italy; c YDA – Institute for Clinical Immunotherapy and Advanced Biological Treatments, Pescara, Italy; d National Research Council of Italy (IFC-CNR), Clinical Physiology Institute, Pisa, Italy; e Department of Clinical and Experimental Medicine, Unit and School of Allergy and Clinical Immunology, University of Messina, Messina

**Keywords:** SARS-CoV-2, HMGB1, IL-1α, IL-33, LL-37, defensins, S100, HSP

## Abstract

The coronavirus SARS-CoV-2, the aetiological agent of COVID-19 disease, is representing a worldwide threat for the medical community and the society at large so that it is being defined as “the twenty-first-century disease”. Often associated with a severe cytokine storm, leading to more severe cases, it is mandatory to block such occurrence early in the disease course, to prevent the patients from having more severe, sometimes fatal, outcomes. In this framework, early detection of “danger signals”, possibly represented by alarmins, can represent one of the most promising strategies to effectively tailor the disease and to better understand the underlying mechanisms eventually leading to death or severe consequences. In light of such considerations, the present article aims at evaluating the role of alarmins in patients affected by COVID-19 disease and the relationship of such compounds with the most commonly reported comorbidities. The conducted researches demonstrated yet poor literature on this specific topic, however preliminarily confirming a role for danger signals in the amplification of the inflammatory reaction associated with SARS-CoV-2 infection. As such, a number of chronic conditions, including metabolic syndrome, gastrointestinal and respiratory diseases, in turn, associated with higher levels of alarmins, both foster the infection and predispose to a worse prognosis. According to these preliminary data, prompt detection of high levels of alarmins in patients with COVID-19 and co-morbidities could suggest an immediate intense anti-inflammatory treatment.

**Key message**
- Alarmins have a role in the amplification of the inflammatory reaction associated with SARS-CoV-2 infection
- a prompt detection of high levels of alarmins in patients with COVID-19 could suggest an immediate intense anti-inflammatory treatment

## Introduction

The coronavirus SARS-CoV-2 is the aetiological agent of COVID-19 disease [1]. This infection is a candidate for being “the twenty-first-century disease”, as it holds the entire earth population in thrall in many ways and it faces the world with unprecedented problems [2].

Most of the patients had mild symptoms in the initial phase of the disease, including cough, mild fever and muscle soreness. On the other hand, some of these subjects developed a more detrimental illness, characterized by acute respiratory distress syndrome (ARDS) and multiple organ failure [3]. Cytokine storm is thought to be the major event causing this unlucky progression [4]. As a consequence, immediately blocking these mediators became of fundamental importance in order to prevent the worsening of the disease and saving patients’ lives [5]. The virus selectively attacks respiratory epithelial cells, macrophages, type II pneumocytes, pericytes and muscle cells, resulting in multiple organ damage, with particular severity in subjects with pre-existing comorbidities [6]. Damaged cells release alarmins, also known as damage-associated molecular patterns (DAMPs), that act as danger signals and promote and exacerbate the inflammatory response.

Alarminsare a group of proteins characterized by high heterogeneity and different functions. After cell damage, they are released in the nearby environment with multiple effects, such as activating innate immunity and recruiting/activating antigen-presenting cells (APC), that in turn stimulate the adaptive immune response with the ultimate action of boosting a huge pro-inflammatory activity [7]. Therefore, the evaluation of alarmins is essential for the compression of inflammatory processes and they are promising biomarkers of clinical conditions with underlying inflammation [8].

From this point of view, alarmins, like interleukin-33 (IL-33), high-mobility group box 1 (HMGB1), antimicrobial peptide LL-37, interleukin 1 alpha (IL-1α), defensin, heat-shock protein (HSP), S100 protein (S100) and calprotectin could play a key role in the COVID-19 and that their study can be useful in the clinical evaluation and follow-up of subjects with the most severe pathology such as those with comorbidities. We speculate a link between the most severe SARS-CoV-2 infections and these danger signals that seem so important in inducing inflammation. Furthermore, the evaluation of alarmins in specific subjects, particularly in those affected by comorbidities, during COVID-19, could be a useful prognostic factor influencing our therapeutic approach.

## Methods

This review was conducted using PubMed and Google Scholar databases. On these search engines, we entered the key terms: SARS-CoV2 , COVID-19, HMGB1, S100, IL-31, IL-33, alarmins and immune system. All the articles dealing with the alarmin in SARS-Cov2 infection have been selected when published in English, in peer-reviewed journals, both as research or review paper. The entire article was read if the abstract indicated the article potentially met the inclusion criteria. Articles were excluded by title, abstract or full text for irrelevance to the topic in question.

### SARS-CoV-2 and immune system

**Figure 1.**
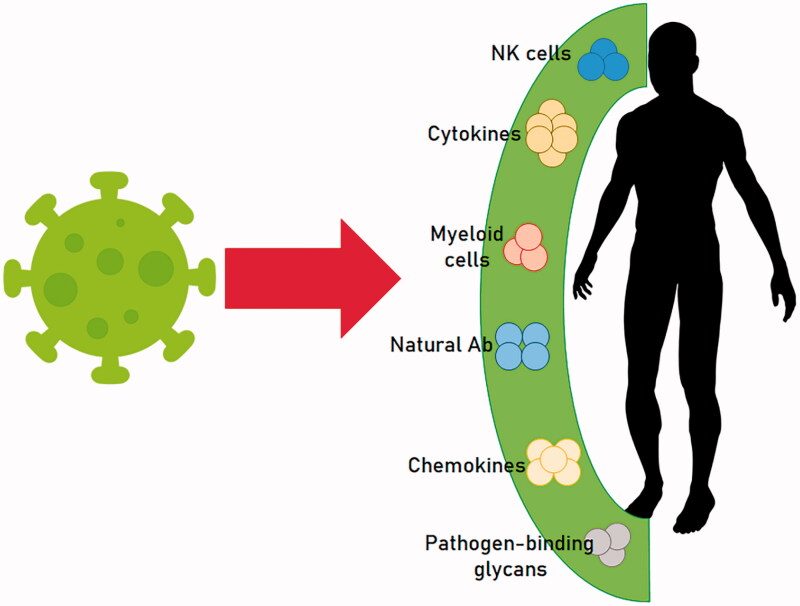
SARS-CoV-2 and “Alarmins”.

Innate immunity has a main role in COVID-19 progression (Figure 1) [9]. Natural killer cells (NK), γδ-T cells and cells of myeloid origin, together with complement and coagulation system, natural antibodies, cytokines, chemokines and pathogen-binding glycans represent the antiviral innate immune defense [10,11]. In particular, type 1 interferons act as potent antivirals as they amplify their own expression and activate NKs cytotoxicity [12,13]. SARS-COV-2 pathogenicity is also determined by viral activation of cytoplasmic NOD-like receptor family, pyrin domain containing 3 (NLRP3) inflammasome, which influences SARS-COV-2 virulence [14]. Interleukin (IL)-1β and IL-18 are, in fact, released by inflammasome stimulation in epithelial, endothelial cells and macrophages. This phenomenon is responsible for a pro-inflammatory response, which determines the severity of COVID-19 symptoms [15]. Furthermore, the viral RNA binding to the toll-like receptor (TLR)3, TLR7, TLR8, and TLR9 promotes the NF-κB pathway and an elevated amount of pro-inflammatory mediators responsible for the virus-induced pro-inflammatory response [14]. There is little data on the other innate immune responses in addition to the high levels of acute-phase reactants and cytokine overgrowth. At present, most research focuses only on severe outcomes and adaptive immune responses. Whereas, innate and acquired immune responses interact during the disease course and in particular at the onset of the critical phases of the disease [5]. In fact, the involvement of the various part of the immune system, with activation of inflammatory molecules, cytokines and mediators imbalance, as well as leukocytes and lymphocytes subpopulation has been demonstrated in SARS-CoV-2 infection [16].

Cellular processes, such as autophagy [17], cytokine storm activation [18], cellular damage [19] and apoptosis [20] induced by SARS-CoV-2 virus seem strictly associated with the production and release of alarmins.

In fact, alarmins, damage-associated molecular patterns (DAMPs), constitute a family of molecules released by cells under stress conditions following infections or sterile insults and have the function of danger signals triggering and augmenting inflammation. Moreover, SARS-CoV-2 requires angiotensin-converting enzyme (ACE) II receptors for its cellular entry and replication. The reduction in ACE II induced by the virus could, in turn, increase alarmins (i.e. HMGB1, IL-31, IL-33, S100), worsening “cytokine storm” and COVID-19 symptoms [21].

These effects are particularly enhanced in patients with comorbidity such as lung fibrosis [22], obesity, non-alcoholic fatty liver disease [23], diabetes [24] in which alarmins play an important role.

Given the importance of these proteins in cell damage-induced inflammation, we summarize and discuss in this review literature data on the alarmins’ role in patients affected by COVID-19 with comorbidities.

### Nuclear alarmins in COVID-19: HMGB1, IL-1α, and IL-33

HMGB1 is a chromatin-linked, non-histonic protein of small dimensions, exerting mediator activity with nuclear, cytosolic, and extracellular functions. Its ligands are chromosomal DNA, Toll-like receptor 3 (TLR3), TLR4, and the receptor for advanced glycation end products (RAGE), in turn triggering nuclear factor (NF)-*κ*B, that up-regulates leukocyte adhesion molecules, proinflammatory cytokines and angiogenic factors, ultimately resulting in inflammation [25]. Its role was certified in several conditions, like obesity [26], insulin resistance and diabetes [27], but also in lung diseases [28], autophagy [29] and hypertension [30].

Some HMGB1 gene polymorphisms were interestingly associated with hypertension in Chinese population, proposing its role in the outcome and course of COVID-19 for some individuals [31]. Wei et al. [32] observed that HMGB1 knockout protected cells from SARS-CoV-2-induced death, and the degree of protection was correlated to HMGB1 levels. Moreover, they demonstrated that HMGB1 acted after viral entry in the cells.

Chen et al. [33]showed that patients’ HMGB1 levels at admission were positively linked to peak CT score and oxygen demand, which is indicative of the severity of acute lung injury and ARDS. The levels of HMGB1 were considerably associated with the COVID-GRAM risk scores.

A recent study reported the augmented immune response of subjects affected by coronavirus through the excessive action of T-cells and CD14+ and CD16+ monocytes, provoking cytokine over-expression. In particular, the IL-1α alarmin was higher during viral infections like H7N9 (flu virus), Covid-19-S (SARS) and Covid-19-M (MERS) [34].

In another study, Chen et al. highlighted that HMGB1 or RAGE inhibitors can prevent SARS-CoV-2 infection by limiting the upregulation of ACE2. In particular, the authors suggested that HMGB1 may be involved in COVID-19 through at least two mechanisms: one is TLR4-mediated cytokine storm in immune cells, and the other is RAGE-mediated ACE2 expression in alveolar epithelial cells [35].

IL-33, initially classified as a cytokine produced by epithelial cells capable of inducing type 2 immune responses, was later identified as an alarmin expressed by a wide variety of cell types, including fibroblasts, mast cells, dendritic cells, macrophages, osteoblasts, endothelial cells, and epithelial cells. Eosinophilia, a marker of type 2 immunity, is frequently reported during SARS-CoV-2 infections. COVID-19 can be characterized by acute events such as pneumonia and acute respiratory distress syndrome, as well as by long-term damages to lungs, like lung remodelling and fibrosis, these last phenomena being often associated with IL-33 as it has been reported by several authors, who showed a link between IL-33 and lung injury, pulmonary viral infections, and chronic lung diseases [36,37]. These observations suggest that IL-33 could represent the main player in COVID-19 pathogenesis. However, IL-33 can also stimulate antiviral cytotoxic T cell activity and antibody production and release. Some authors speculate that the persistence of cells producing IL-33 in response to T cell activation may give some benefits in the case of later contact with the virus, further linking IL33 to the COVID-19 immunobiology[38].

### Granule-derived alarmins in COVID-19: LL-37 and defensins

LL-37 is a 37 amino acid peptide, resulting from the cleavage of an 18 kDa polypeptide, hCAP18, and is also the only anti-microbial peptide (AMP) belonging to the cathelicidin family in humans. It is produced by epithelial cells of the skin and the respiratory tract, by NK cells, B cells, mast cells [39,40].

There are reports on the protective effect of LL-37 from SARS-CoV-2 infection. It is known that SARS-CoV-2 requires ACE2 receptors for its cellular entry and replication. In fact, the receptor binding domain (RBD) of the Spike protein interfaces with the N-terminal helix (NTH) of the peptidase domain of ACE2. The receptor-binding motif (RBM) within the RBD, accepts the NTH. [41]. Some research shows the binding of LL-37 to the RBD of SARS-CoV-2 and LL-37 structural alignment to the NTH could stop RBD from binding ACE2 by inhabiting the space intended by the virus for NTH. This result suggests that LL-37 is protective and that the use of Vitamin D, able to stimulate the production of LL-37 by epithelial cells, can be a therapeutical means to counteract SARS-CoV-2 [42].

COVID-19 also provokes gastrointestinal symptoms. For this reason, cas001, an oral anti-viral agent was elaborated, being capable of blocking the action of LL-37 and sustaining the probiotic action of *Lactococcus lactis.* Cas001 challenges the symptoms and fosters nucleic acid clearance in patients with mild symptoms, especially gastrointestinal ones. This report suggests its use for the treatment of patients with mild symptoms as a support drug or as a single therapy for clinical applications [43].

HD5, the most abundant alfa defensin secreted by intestinal Paneth cells, is in contact with ACE2 on the membrane of enterocytes. It has been shown that there is a structure-dependent interaction between HD5 and ACE2 and that the binding of HD5 to ACE2 cloaked several sites in its ligand-binding domain, among which Asp30 and Lys31, that are crucial for SARS-CoV spike to bind ACE2. Accordingly, SARS-CoV-2 S1 binding and S pseudovirions entry to enterocytes were inhibited by HD5 [44]. Patients receiving small intestine transplantation or suffering from inflammatory bowel diseases such as Crohn’s disease might be more susceptible to SARS-CoV-2 than healthy individuals being them characterized by HD5 deficiency [45].

### Cytoplasmatic alarmins in COVID-19: S100 and HSP

The most abundant alarmins in many inflammatory disorders, S100A8 (myeloid-related protein 8/MRP8) and S100A9 (MRP14), belong to the group of S100-proteins, defining a family of small (molecular weight of about 10–12 kDa) calcium-binding molecules. Members of this S100-protein family are characterized by a tissue or cell type-specific expression pattern [46]. S100A8 and S100A9 exist mainly together as a biologically functional heterodimer known as S100A8/A9 or calprotectin. Calprotectin is found in abundance in neutrophils, where it can account for almost two-thirds of soluble protein in the cytosol. Calprotectin may also be detected at low levels in monocytes, macrophages, platelets, and squamous epithelial cells. Upon neutrophil activation or death, calprotectin is released extracellularly, where it has microbicidal functions (*via* heavy-metal chelation) and also serves as a pro-inflammatory ligand for innate receptors, such as receptor for advanced glycation end-products (RAGE) and Toll-like receptor 4 (TLR4) [47]. A recent study reported markedly elevated levels of serum and plasma calprotectin in the majority of patients hospitalized with COVID-19. The authors raise the need to investigate the specific form of neutrophil activation and/or cell death that floods COVID-19 blood with excessive calprotectin. Tissue damage and neutrophil necrosis are potential sources of passive calprotectin release.

The realization that the levels of S100A8/A9 were significantly higher in the most severe patients [33] is of great clinical significance as it allows for the early identification of COVID-19 patients who may be admitted to ICU or facing death. Furthermore, COVID-19 patients treated in general wards had a significantly elevated levels of S100A8/A9, but not HMGB1 as compared to healthy controls, suggesting that S100A8/A9 is a more sensitive alarmin than HMGB1 in response to SARS-CoV-2 infection. Furthermore, a positive correlation between circulating levels of calprotectin and growth differentiation factor 15 (GDF-15, an emerging inflammatory biomarker), that is significantly higher in COVID-19 patients passing away, is even more in support of their importance in the assessment of prognosis in COVID-19 patients [48].

In addition to being an inflammatory marker, calprotectin may also have a direct role in the self-amplifying thrombo-inflammatory storm of COVID-19 *via* engagement and activation of innate immune sensors such as RAGE and TLR4 [49].

More scientific evidence showed the close correlation between faecal calprotectin and COVID-19, in particular, in patients with COVID-19 associated diarrhoea, elevated FC (largely expressed by neutrophil granulocytes) and systemic IL-6 response. These data supported the notion that SARS-CoV-2 infection exerts gut tropism characterized by an acute inflammatory response that potentially deteriorates the course of human IBD [50], even if there are no definite evidence, furthermore, it is unknown whether immunosuppressive treatment of IBD affects the susceptibility to (or the course of) COVID-19.

S100B, a calcium-binding protein, a reliable biomarker in inflammatory disorders having RAGE as the main receptor, was evaluated as a peripheric marker of inflammation in a group of patients admitted for COVID-19. Elevated levels of the alarmin were, in particular, associated with kidney and liver damage with ALT and creatinine levels augmented too, suggesting a more susceptibility of subjects affected by hypertension and metabolic diseases [51].

Hsp90 family alarmin, among the most abundantly expressed molecular chaperones, is required for the replication of numerous viruses, including DNA viruses, double-stranded RNA viruses, positive- and negative-stranded RNA viruses. It has been demonstrated that the Hsp90 phosphorylates mTOR to facilitate the translation of viral mRNA. The central role of Hsp90 for SARS-CoV-2 propagation was highlighted [51] and correspondingly Hsp90 inhibitors have been shown useful in treating SARS-CoV-2 infection [50,52].

**Figure 2.**
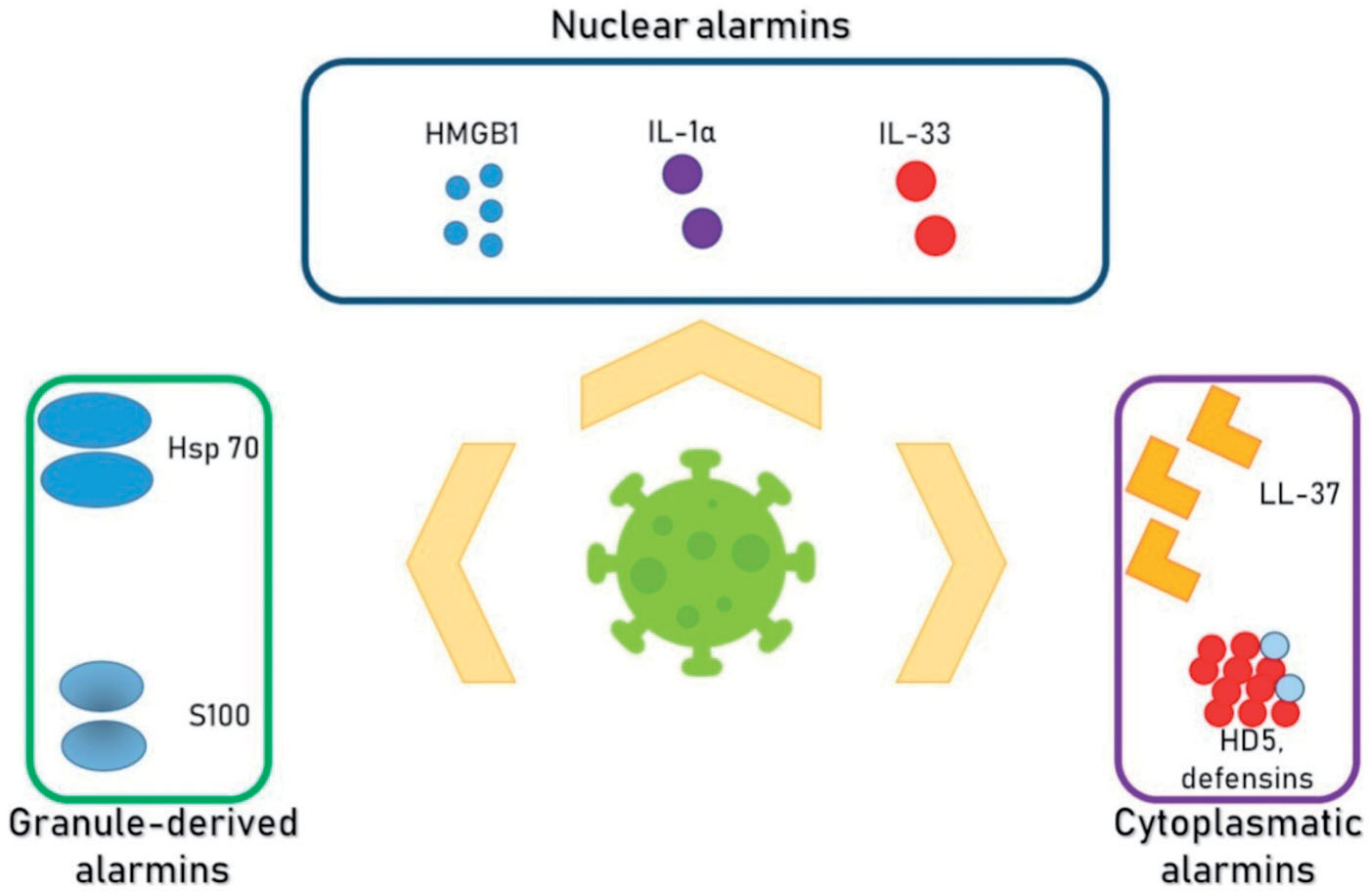
Metabolic syndrome, chronic inflammation, alarmins and COVID-19.

An overall view of the alarmins enhanced within SARS-CoV-2 is displayed in Figure 2.

## Discussion

During the last decade, many authors focussed on the individuation of an always increasing quantity of alarmins in inflammatory diseases. HMGB-1, S100, IL-33 are only some examples of how these danger signals are important in the etiopathogenesis of pathologies like heart failure, obesity, diabetes, hypertension. The authors often suggested that the presence of a high inflammatory status influences cellular homeostasis resulting in an impaired organ function causing or favouring the disease [52–54].

It is well known that some patients are particularly sensitive to SARS-COV-2 infection and COVID-19 spread, while some have no or mild symptoms and others develop the most severe disease. NK cell, γδ-T cells and cells of myeloid origin were confirmed having a key role in the chemokine and cytokine storm development, that is associated with severe COVID-19, but data are emerging on the activation of other amplifying pathways and triggers. A potential proinflammatory role could be due also to type 1 interferons that, in the attempt of controlling virus diffusion by activating NK, could fail their self-limiting ability *via* STAT1-dependent mechanisms [12]. On the other side, TRLs, which activated the NFkB cascade and the inflammasome intervention through IL-1β and IL-18 are only some tiles of the mosaic. COVID-19 most severe forms develop in patients with comorbidities such as metabolic syndrome, obesity, fatty liver diseases, hypertension [21,26,41,53]. All these conditions are characterized by chronic inflammatory status often matched with high alarmins levels (Table 1) [34].

**Table 1.** Enhanced alarmins in COVID-19 and relationship with associated comorbidities.

Alarmins	Target	Comorbidity	Ref
HMGB1 (nuclear)	Monkeys	Not evaluated	[32]
HMGB1 (nuclear)	Humans	Not evaluated	[33]
HMGB1(nuclear)	Humans	Acute respiratory distress syndrome, sepsis, septic shock	[35]
IL-1α(nuclear)	Humans	Not evaluated	[34]
IL-33(nuclear)	Humans	Not evaluated	[36]
LL-37 (granule-derived)	*In vitro*	Not evaluated	[40]
LL-37(granule-derived)	Mice, rats, Humans	IBD	[41]
Defensin 5 (granule-derived)	*In vitro*	IBD	[42]
Calprotectin (cytoplasmatic)	Humans	Respiratory diseases IBD hypertension, non-asthma respiratory diseases, diabetes mellitus, immunosuppression, cardiovascular disease, chronic kidney disease	[47,48]
S100 (cytoplasmatic)	Humans	Not evaluated	[33]
Hsp 90 (citoplasmatic)	Humans	Not evaluated	[57]

**Figure 3.**
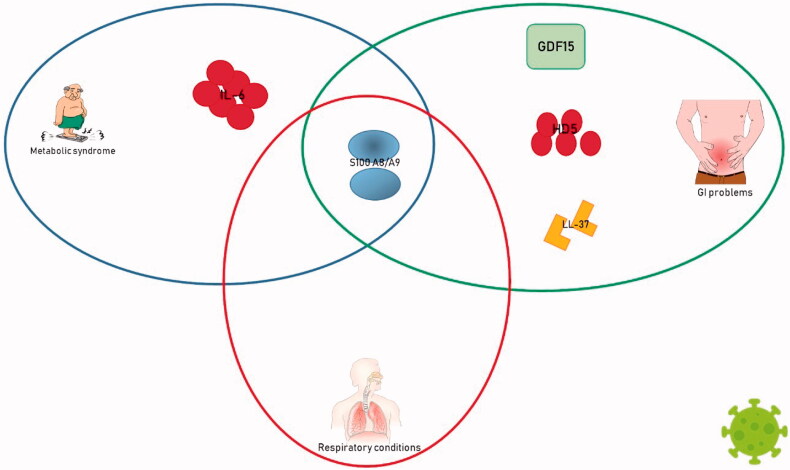
Enhanced alarmins in SARS-CoV-2 classified upon family.

In fact, patients with COVID-19 had augmented values of HMGB-1 and IL-33 while facing lung injury or fibrotic processes. Both these alarmins and IL-1α were intimately linked to an intense inflammatory response. LL-37 had a double-face behaviour in SARS-CoV-2 infection, probably linked to disease severity and stage. However, also other organs than lungs were observed as being susceptible to the infection. The gut was not an exception and the defensin HD5, the calprotectin S100A8/A9 and the GDF-15 had a specific role both in the gastroenterological manifestation and the prognosis of COVID-19 (Figure 3).

However, there are some limitations in this review, mainly due to the novelty of the disease and to the little known about alarmins. In fact, these danger signals aroused attention only in the last decade and COVID-19 was firstly described in the last months of 2019.

Data on alarmins in COVID-19 disease are still limited but the preliminary research confirmed danger signals’ role in the amplification of the proinflammatory reaction. In this scenario, some chronic diseases such as hypertension, obesity, diabetes demonstrated to both foster the infection and predispose to a worse prognosis. Therefore, testing qualitative and quantitative alarmin levels could help detecting subjects at risk and evaluating in advance disease course severity. Further studies both on comorbidity incidence and on alarmins mechanisms of action during SARS-CoV-2 infection could help better understanding such condition. The most recent research particularly focussed on the association among peripheric alarmins, cytokines levels and disease severity indicate the potential for alarmins as therapeutical tools/target for COVID-19. Experimental data on animal models and in some human trials demonstrated their potential in blocking inflammation (i.e. anti-IL-33 antibodies) [55,56]. These promising results in using alarmins inhibitors could be effective in contrasting the cytokine storm responsible for most of the multiorgan damage. The next step should be focussed on testing diverse alarmin inhibitors in several pro-inflammatory conditions. Moreover, the presence of alarmins during COVID-19 infection was confirmed as being as detrimental as in other inflammatory internal diseases due to their pro-inflammatory action. Results suggest that dosing alarmins in patients with SARS-CoV-2 infection and co-morbidities could help in the early identification of the most severe forms, so allow starting more intense anti-inflammatory treatment. Starting this treatment as soon as possible could lead to a better prognosis. Further studies, concerning the role of every alarmin analyzed both singularly and simultaneously in patients affected by COVID-19 could be useful. Considering the presence of co-morbidities in SARS-CoV-2 infected patients together with the presence of a danger signal could be the key for next advances.

## Data Availability

Data are taken from previously published articles since this is a review of the current literature to date.
